# Bioinformatics analysis of the clinical relevance of CDCA gene family in prostate cancer

**DOI:** 10.1097/MD.0000000000028788

**Published:** 2022-02-04

**Authors:** Peng Gu, Dongrong Yang, Jin Zhu, Minhao Zhang, Xiaoliang He

**Affiliations:** a Department of Urology, Xishan People's Hospital of Wuxi City, Wuxi, Jiangsu, China; b Department of Urology, Second Affiliated Hospital of Soochow University, Suzhou, Jiangsu, China.

**Keywords:** bioinformatics analysis, cell division cycle associated gene, prostate cancer

## Abstract

**Background:**

Prostate cancer (PCa) is the second most frequent cancer in men worldwide, and its mortality rate is increasing every year. The cell division cycle-associated (CDCA) gene family plays vital roles in the cell cycle process, but an analysis of these proteins in PCa is still lacking.

**Methods:**

UALCAN and GEPIA were used to examine the transcriptional data and survival of the CDCA gene family in PCa patients. CDCA genetic alterations, prognostic value of genetic alterations, and correlations of CDCAs with each other in PCa were downloaded from cBioPortal. The functional enrichment data of CDCA-related genes were analyzed using DAVID.

**Results:**

Six CDCA genes were upregulated in PCa tissues relative to those in normal tissues (*P* < .001), including NUF2, CDCA2, CDCA3, CDCA5, CBX2, and CDCA8. The expression levels of the 6 CDCAs were related to the tumor Gleason score (*P* < .05). In addition, survival analysis using GEPIA suggested that PCa patients with increased NUF2, CBX2, and CDCA2/3/5/8 expression levels had poor relapse-free survival (*P* < .05). Distinct patterns of genetic alterations of the 6 CDCAs were observed in PCa, and pairwise comparison of the mRNA expression of the 6 CDCAs displayed a close relationship. The biological functions of CDCA-related genes are principally associated with the activation of the following pathways: cell cycle, Fanconi anemia pathway, microRNAs in cancer, oocyte meiosis, and homologous recombination.

**Conclusions:**

Upregulated CDCA (NUF2, CBX2, and CDCA2/3/5/8) expression in PCa tissues may play a crucial role in the occurrence of PCa. These CDCAs can predict relapse-free survival prognosis and the Gleason score of patients with PCa. Moreover, CDCAs probably exert their functions in tumorigenesis through the cell cycle and miRNAs in the cancer pathway.

## 1 Introduction

Prostate cancer (PCa) is a common urogenital cancer, with an estimated 248,530 new cases and 34,130 deaths in the United States by 2021.^[1]^ PCa is the second leading cause of cancer-related death in American men, and survival rates are low for PCas that advance to metastatic castration-resistant prostate cancer (CRPC). Therefore, it is necessary to study the underlying mechanisms of tumorigenesis and the development of PCa, and to identify highly sensitive and specific tumor-related biomarkers.

The family of cell division cycle associated (CDCA) proteins has 8 members: CDCA1 (also known as NUF2), CDCA2, CDCA3, CDCA4, CDCA5, CDCA6 (also known as CBX2), CDCA7, and CDCA8. Interestingly, although they belong to different complexes, they collaborate during separation and throughout the cell cycle, including during cell division and other biological activities.^[2]^ Previous studies and integrated analyses have revealed that some members of the CDCA gene family may be overexpressed in pancreatic cancer,^[3]^ ovarian cancer,^[4]^ clear cell renal cell carcinoma,^[2]^ endometrial carcinoma,^[5]^ lung carcinoma,^[6]^ hepatocellular carcinoma,^[7]^ breast cancer,^[8]^ and head and neck squamous cell carcinoma.^[9]^ However, the function of this gene family in PCa has not been systematically analyzed.

In this study, we used several online networking tools to assess the role of each CDCA member in PCa. First, we analyzed the expression levels of each CDCA member in cancer and normal tissues. We also analyzed the relationship between the identified upregulated CDCAs and PCa survival and Gleason score. Then, CDCA genetic alterations and their prognostic value and correlations of CDCAs with each other in PCa were investigated. Finally, we predicted the specific function of CDCAs in PCa.

## 2 Materials and methods

### 2.1 UALCAN analysis

UALCAN (http://ualcan.path.uab.edu/) is a website that helps analyze, integrate, and discover cancer transcriptomic data and perform deep analyses of The Cancer Genome Atlas (TCGA) gene expression information.^[10]^ This enabled us to provide differential expression analyses of PCa and normal prostate tissues, as well as to obtain the profiling of tumor Gleason score.

### 2.2 Survival analysis by GEPIA

GEPIA (http://gepia.cancer-pku.cn/) is a web server that analyzes RNA expression based on data from TCGA and the Genotype-Tissue Expression project.^[11]^ In the survival analysis, each median expression of log10 (transcripts per million) of CDCAs was set as the cutoff to divide the patients into high- and low-expression groups. *P* value < .05 was set as the cut-off criterion.

### 2.3 TCGA data and cBioPortal

cBioPortal (http://www.cbioportal.org/) for cancer genomics provides comprehensive analyses of complex tumor genomics and clinical profiles from TCGA.^[12]^ We used this tool to analyze genomic alterations in CDCAs in PCa. The prostate adenocarcinoma (TCGA, Firehose Legacy) dataset, including data from 499 cases with pathology reports, was selected for further analysis of CDCAs. Spearman correlations of CDCAs with each other and the impact of CDCA alterations on PCa patient survival were also downloaded from cBioportal.

### 2.4 Genes correlated with CDCAs and related pathways

Genes correlated with NUF2, CBX2, and CDCA2/3/5/8 in PCa samples were downloaded from UALCAN, with the thresholds set as R ≥ 0.5 and *P* value < .05. The final CDCA-related genes were defined as genes that overlapped in all 6 gene sets. A Venn diagram was constructed using an online web tool (http://bioinformatics.psb.ugent.be/webtools/Venn/). Gene enrichment was annotated according to the gene ontology (GO) molecular functions, GO biological processes, GO cellular components, and Kyoto Encyclopedia of Genes and Genomes (KEGG) pathways using DAVID (https://david.ncifcrf.gov/). *Statistical significance* was set at *P* < .05.

### 2.5 Ethical statement

All data in this study were obtained from open public databases; we did not obtain these data from patients directly or intervene in these patients. Therefore, ethical approval was not required for this study.

## 3 Results

### 3.1 Expression levels of CDCAs in patients with PCa in TCGA database

**Figure 1:**
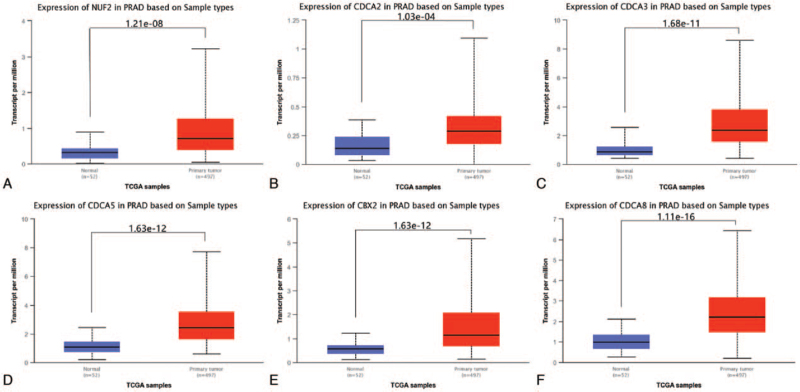
Transcriptional expression levels of 6 CDCAs in PCa and normal prostate tissues (TCGA database, *P* < .001). (A) NUF2, (B) CDCA2, (C) CDCA3, (D) CDCA5, (E) CBX2, (F) CDCA8. CDCAs = cell division cycle associated genes, PCa = prostate cancer, TCGA = The Cancer Genome Atlas.

We first used TCGA database from the UALCAN website to compare the expression levels of CDCAs between PCa and normal prostate tissues. It contained 497 PCa tissue samples and 52 normal prostate samples. As shown in Figure 1, 6 CDCAs, including NUF2, CDCA2, CDCA3, CDCA5, CBX2, and CDCA8, were significantly upregulated in PCa tissues compared to normal prostate tissues (*P* < .001).

### 3.2 Correlation between CDCAs transcriptional expression levels and Gleason score

**Figure 2:**
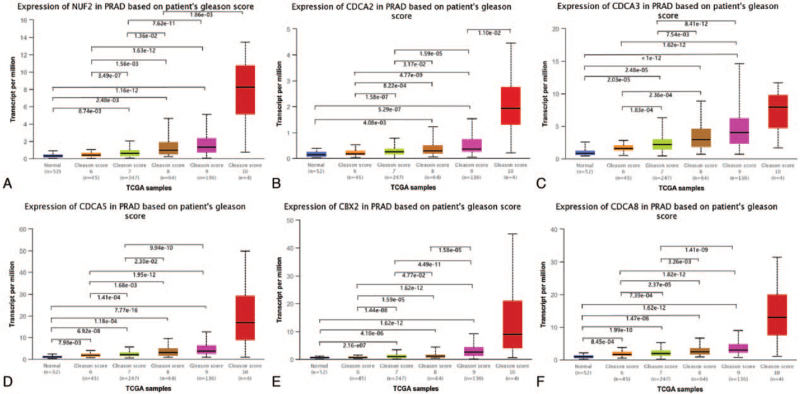
Correlation between the transcriptional expression level of each CDCAs and Gleason score (TCGA database, *P* < .05). (A) NUF2, (B) CDCA2, (C) CDCA3, (D) CDCA5, (E) CBX2, (F) CDCA8. CDCAs = cell division cycle associated genes, TCGA = The Cancer Genome Atlas.

Then, the effect of the transcriptional expression level of each member of the 6 CDCAs on the tumor Gleason score was investigated. As Figure 2 shows, the upregulated expression levels of NUF2, CBX2, and CDCA2/3/5/8 (Gleason score 7/8/9 vs Gleason score 6 and Gleason score 8/9 vs Gleason score 7, both *P* < .05) significantly matched the more advanced Gleason score.

### 3.3 Prognostic value of CDCAs mRNA levels in PCa patients

**Figure 3:**
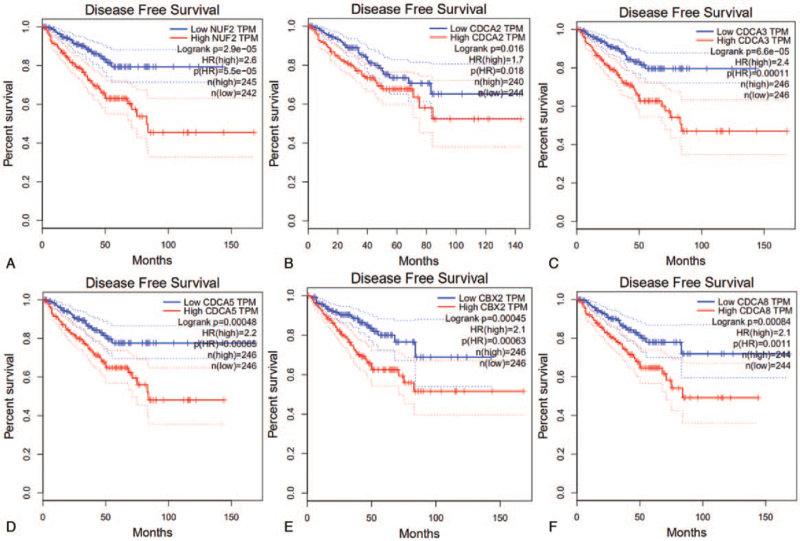
Correlation between 6 CDCAs expression levels and RFS in PCa patients (GEPIA, *P* < .05). (A) NUF2, (B) CDCA2, (C) CDCA3, (D) CDCA5, (E) CBX2, (F) CDCA8. CDCAs = cell division cycle associated genes, RFS = relapse-free survival, PCa = prostate cancer.

Survival analysis was based on GEPIA data. In the present study, all 6 CDCA mRNA levels were associated with relapse-free survival (RFS) in PCa patients (*P* < .05), but not with overall survival (data not shown). NUF2 had the highest hazard ratio (HR) of 2.6 that ranked the top. High expression levels of CDCA2 (HR = 1.7), CDCA3 (HR = 2.4), CDCA5 (HR = 2.2), CBX2 (HR = 2.1), and CDCA8 (HR = 2.1) were associated with poor disease-free survival (Fig. 3).

### 3.4 CDCAs genetic alterations in PCa

**Figure 4:**
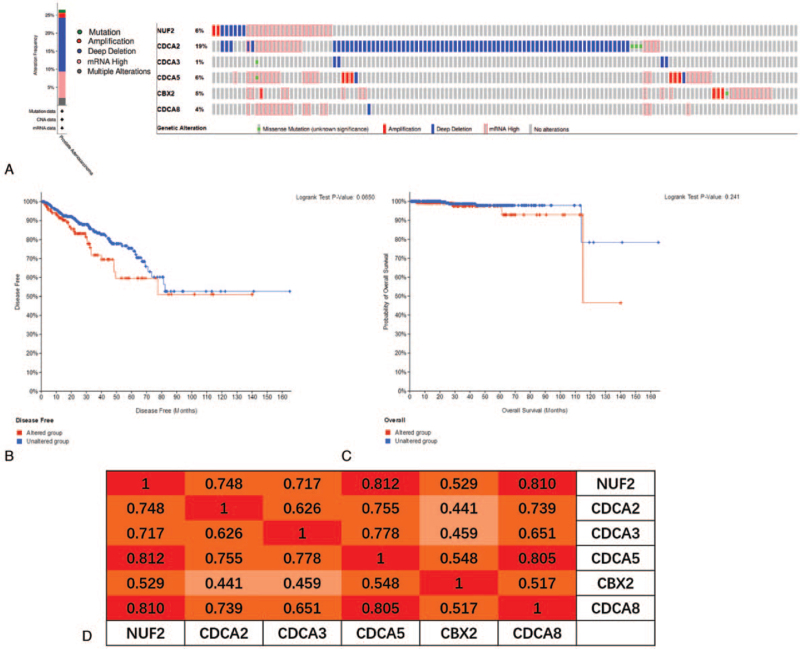
CDCAs genetic alterations and its prognostic value, and correlations of 6 CDCAs with each other in PCa (cBioPortal). (A) OncoPrint visual summary of variations on query of 6 CDCAs. (B, C) The 6 CDCAs genetic alterations did not impact the disease free and overall survival rates of PCa patients (*P* values, .065 and .241, respectively). (D) Spearman correlations between different CDCAs expression levels in PCa (*P* = .000). CDCAs = cell division cycle associated genes, PCa = prostate cancer.

To gain in-depth insight into the molecular mechanisms of differential expression of the 6 CDCAs, genetic alterations were analyzed in PCa patients. Alterations were detected in 27% of the PCa samples using the OncoPrint visual summary (Fig. 4A). CDCA2 had the highest probability of alterations (19%), followed by NUF2 and CDCA5 (both 6%). Generally, deep deletions account for the majority of alterations. Patients with genetic alterations in the 6 CDCAs did not show different disease-free and overall survival rates compared to those without alterations (*P* = .065 and *P* = .241, respectively) (Fig. 4B, C). We also calculated the correlations between the 6 CDCAs by analyzing their mRNA expression (RNA sequencing [RNA-seq] version (v.)2 RSEM). Spearman correlation analysis results indicated significant and positive correlations among all 6 CDCAs (*P* = .000, Fig. 4D).

### 3.5 Functional enrichment of CDCAs-related genes

**Figure 5:**
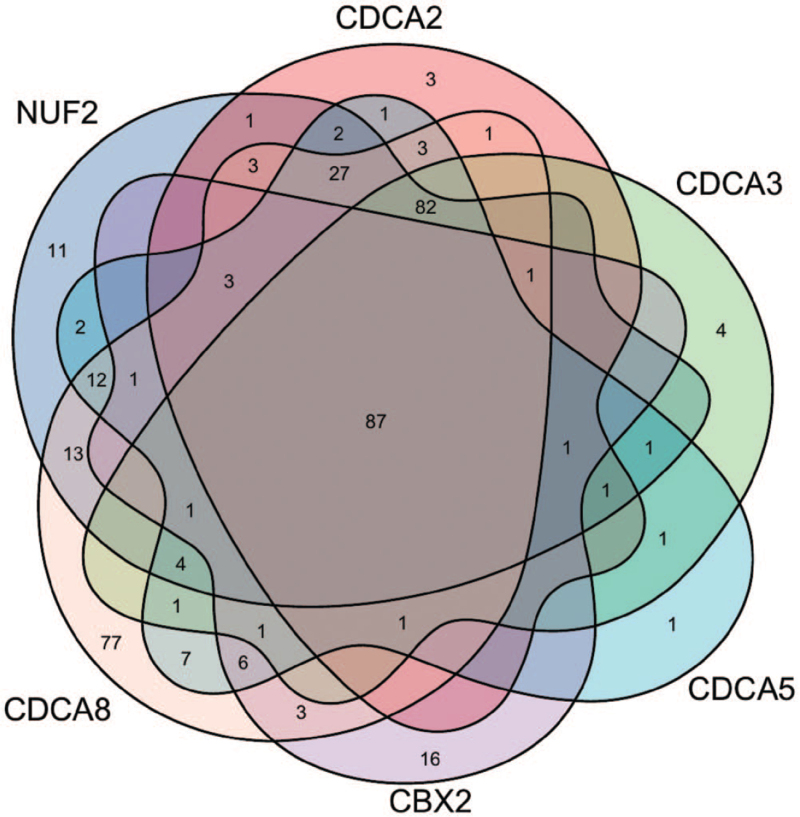
The 87 overlapping genes that all correlated with 6 CDCAs displayed in a Venn diagram. CDCAs = cell division cycle associated genes.

To explore the biological classification of the 6 CDCAs, we first identified genes correlated with CDCAs from ULCAN (R ≥ 0.5, *P* < .05). In addition, 253, 217, 186, 246, 121, and 334 genes that correlated with NUF2, CDCA2, CDCA3, CDCA5, CBX2, and CDCA8, respectively, were selected. Specifically, we identified candidates that overlapped in the 6 gene sets by drawing a Venn diagram. Finally, 87 overlapping genes were associated with all 6 CDCAs (Fig. 5), and the gene list is shown in Table 1. Functional and pathway enrichment analyses were then performed using DAVID. GO function analysis revealed enrichment of 87 overlapping genes and 6 CDCAs in functions related to the nucleus, nucleoplasm, cytoplasm and cytosol, protein binding, ATP binding, and DNA binding, which participate in cell division, mitotic nuclear division, and sister chromatid cohesion. KEGG pathway analysis indicated that these genes were mainly enriched in the cell cycle, Fanconi anemia pathway, microRNAs in cancer, oocyte meiosis, and homologous recombination. The 10 most enriched classes based on GO function analysis and the 5 most enriched KEGG pathways are listed in Table 2.

**Table 1:** Eighty-seven overlapping genes correlated with all the 6 CDCAs from ULCAN (R ≥ 0.5; *P* < .05).

Names	Number of correlated genes	87 overlapping genes
NUF2	253	ARHGAP11A, ASF1B, AURKA, AURKB, BIRC5, BLM, BRCA1, BUB1, BUB1B, CCNA2, CCNF, CDCA4, CDCA8, CDC20, CDKN3, CENPA, CENPE, CENPK, CIT, C9orf100, C16orf75, DNMT3B, DONSON, DSCC1, DTL, EME1, EPR1, EZH2, E2F1, FAM64A, FAM72B, FAM111B, FANCA, FANCD2, FANCI, GINS1, GINS4, HJURP, HMGB3, HN1, IQGAP3, KIAA0101, KIFC1, KIF2C, KIF4A, KIF15, KIF20A, KIF23, KNTC1, LOC100128191, MELK, MKI67, MND1, MYBL2, NCAPG, NFKBIL2, NUSAP1, OIP5, ORC6L, PBK, PIF1, PKMYT1, PLK1, POC1A, PRC1, PTTG1, RACGAP1, RAD51AP1,RAD54B, RAD54L, RFC4, SGOL2, SKA1, SPAG5, SPC24, SPC25, STMN1,TACC3, TCF19, TK1, TPX2, TRIP13, TROAP, UBE2T, UHRF1, ZNF695, ZWINT
CDCA2	217	
CDCA3	186	
CDCA5	246	
CBX2	121	
CDCA8	334	

CDCAs = cell division cycle associated genes.

**Table 2:** GO and KEGG pathway analysis of 6 CDCAs and CDCAs-related genes.

	Category	Term	Count	%	*P* value
GO analysis	GOTERM_MF_DIRECT	GO:0005515∼protein binding	73	85.88	3.11E-13
	GOTERM_CC_DIRECT	GO:0005634∼nucleus	61	71.76	1.34E-15
	GOTERM_CC_DIRECT	GO:0005654∼nucleoplasm	44	51.76	8.48E-15
	GOTERM_CC_DIRECT	GO:0005737∼cytoplasm	42	49.41	5.11E-05
	GOTERM_CC_DIRECT	GO:0005829∼cytosol	34	40.00	3.40E-06
	GOTERM_BP_DIRECT	GO:0051301∼cell division	28	32.94	1.20E-25
	GOTERM_BP_DIRECT	GO:0007067∼mitotic nuclear division	26	30.59	1.24E-26
	GOTERM_MF_DIRECT	GO:0005524∼ATP binding	26	30.59	1.19E-08
	GOTERM_BP_DIRECT	GO:0007062∼sister chromatid cohesion	18	21.18	5.67E-22
	GOTERM_MF_DIRECT	GO:0003677∼DNA binding	18	21.18	0.001893871
KEGG pathway	KEGG_PATHWAY	hsa04110:Cell cycle	8	9.41	3.44E-07
	KEGG_PATHWAY	hsa03460:Fanconi anemia pathway	7	8.24	4.09E-08
	KEGG_PATHWAY	hsa05206:MicroRNAs in cancer	7	8.24	6.92E-04
	KEGG_PATHWAY	hsa04114:Oocyte meiosis	6	7.06	6.08E-05
	KEGG_PATHWAY	hsa03440:Homologous recombination	4	4.71	1.84E-04

BP = biological process, CC = cellular component, CDCAs = cell division cycle associated genes, GO = gene ontology, KEGG = Kyoto Encyclopedia of Genes and Genomes, MF = molecular function.

## 4 Discussion

Malfunction in cell division can lead to cancer progression. Disturbance of cell cycle regulation is an important biological feature of malignant tumors, and can lead to reduced apoptosis, unlimited proliferation, and metastasis in malignant cells. Cell cycle disruption is one of the most important causes of malignant tumors.^[13]^ Numerous cell cycle-related genes are dysregulated in cancer and may be potential targets for drug therapy.^[14]^ There are 8 members of the CDCA gene and protein families, namely CDCA1–8. Not only are they essential for normal cell function, but they also play an important role in the proliferation of cancer cells.

In the present study, we attempted to demonstrate the prognostic value of 8 CDCAs in patients with PCa. First, we compared the gene expression levels of CDCAs in TCGA database and found that NUF2, CDCA2, CDCA3, CDCA5, CBX2, and CDCA8 were upregulated in PCa tissues and that the 6 CDCAs were regarded as risk factors for RFS probability in GEPIA. In the UALCAN analysis, 6 increased CDCAs were observed in the advanced tumor Gleason score. Using the cBioPortal platform, genetic alterations of the 6 CDCAs were observed, and pairwise comparison of the mRNA expression of the 6 CDCAs displayed a close relationship. Genetic alterations may not affect the prognosis of patients with PCa. Genes correlated with NUF2, CBX2, and CDCA2/3/5/8 in PCa samples were downloaded from UALCAN. Finally, 87 overlapping CDCA-related genes were obtained and are displayed in a Venn diagram. We found that the CDCAs were not only enriched in the biological process of the cell cycle but were also enriched in the Fanconi anemia pathway, microRNAs in cancer, oocyte meiosis, and homologous recombination.

CDCA1 was initially identified as a component of the kinetochore complex, which is evolutionarily conserved and important for the stability of kinetochore and microtubule.^[15]^ Depletion of CDCA1 has been reported to lead to a deficiency of kinetochore microtubule attachment and activation of the spindle checkpoint, ultimately leading to the death of mitotic cell.^[16]^ In a study by Zhao et al,^[17]^ CDCA1 was overexpressed in PCa cell lines, and the expression level of CDCA1 in human PCa tissues was significantly higher than that in adjacent normal tissues. They reported that CDCA1 is a promising diagnostic and prognostic biomarker as well as a target for the treatment of PCa. In addition, a clinical trial conducted on patients with CRPC determined that CDCA1 peptide vaccination could induce peptide-specific cytotoxic T lymphocytes in patients with CRPC.^[18]^ CDCA2 is a nuclear protein that binds to protein phosphatase 1γ, which is responsible for the targeting of protein phosphatase 1 to chromatin during anaphase and controls cell proliferation in vitro.^[19]^ Zhang et al^[20]^ found that CDCA2 is overexpressed in PCa and many other cancer types, and that it acts as an oncogene in PCa, which has been demonstrated in in vivo and in vitro studies. CDCA3 is a “trigger” for mitotic entry and has been reported to mediate cell cycle progression.^[21]^ CDCA3 functions as a part of the S phase kinase-associated protein 1/Cullin 1/F-box (SCF) E3 ubiquitin ligase complex to mediate the destruction of the mitosis inhibitory kinase wee1, thus imparting an important effect on the cell cycle.^[22]^ Chen et al^[23]^ suggested that HoxB3 promotes PCa progression by transactivating CDCA3 expression and preventing G1 phase arrest. CDCA5 ensures precise cell chromosome separation during meiosis and mitosis and maintains sister chromatid cohesion by stabilizing the cohesive complex; it also plays an important role in DNA repair.^[24]^ Moreover, CDCA5 regulates the activity of cell cycle-related proteins and transcription factors, thereby promoting proliferation and participating in apoptosis in cancer cells.^[25]^ In PCa, Ji et al^[26]^ elucidated that CDCA5 functions through the ERK signaling pathway to promote tumor progression. CDCA6 maintains the transcriptionally repressed state of many genes throughout development through histone modification and chromatin remodeling.^[2]^ Clermont et al^[27]^ demonstrated CDCA6 was upregulated in androgen-independent and metastatic PCa cells and that increased expression levels predict poor clinical efficacy. Furthermore, CDCA6 depletion induced PCa cell death and proliferation arrest by regulating the expression of a key subset of genes, indicating that CDCA6 may potentially be used as a drug target in CRPC. CDCA8 is a member of the chromosomal passenger complex that is necessary for genome transmission during cell division. It plays a crucial role in mitosis, intersecting chromosome segregation, and cell division in cancers. Studies have revealed that CDCA8 is upregulated in colorectal cancers, and that deficiency of CDCA8 induces apoptosis of cancer cells and suppresses growth.^[28]^ CDCA8 may act as a promoter of lymph node metastasis in PCa and hopefully become a new diagnostic and therapeutic factor for PCa by bioinformatics analysis,^[29]^ but validation is lacking in vivo and in vitro.

## 5 Conclusions

In conclusion, our study sheds light on the clinical significance and potential biological function of the CDCA gene family in PCa. NUF2, CBX2, and CDCA2/3/5/8 are overexpressed in PCa tissues. Six upregulated CDCAs were observed in the advanced tumor Gleason score and may act as risk factors for RFS in patients with PCa. A pairwise comparison of the mRNA expression of the 6 CDCAs showed a close relationship. Although genetic alterations in the 6 CDCAs were observed, they might not affect the prognosis of patients with PCa. Moreover, CDCAs probably exert their functions in tumorigenesis through the cell cycle and miRNAs in cancer.

## Author contributions

PG wrote the manuscript, carried out the research methodology, and acquired the data. DY, JZ, and MZ performed data analysis and provided technical support. DY and XH conceived of and designed the study. All authors have read and approved the manuscript and agreed to be accountable for all aspects of the research.

**Conceptualization:** Peng Gu, Dongrong Yang, Xiaoliang He.

**Data curation:** Peng Gu, Jin Zhu, Minhao Zhang.

**Formal analysis:** Peng Gu, Jin Zhu, Minhao Zhang.

**Funding acquisition:** Xiaoliang He.

**Investigation:** Peng Gu.

**Methodology:** Peng Gu, Jin Zhu, Minhao Zhang.

**Software:** Peng Gu, Minhao Zhang.

**Supervision:** Peng Gu, Dongrong Yang, Xiaoliang He.

**Writing – original draft:** Peng Gu.

**Writing – review & editing:** Dongrong Yang, Xiaoliang He.
